# Exercise Therapy in Nonspecific Low Back Pain among Individuals with Lower-Limb Amputation: A Systematic Review

**DOI:** 10.3390/life13030772

**Published:** 2023-03-13

**Authors:** Agnieszka Wnuk-Scardaccione, Klaudia Zawojska, Marta Barłowska-Trybulec, Agnieszka Irena Mazur-Biały

**Affiliations:** Department of Biomechanics and Kinesiology, Chair of Biomedical Sciences, Institute of Physiotherapy, Faculty of Health Sciences, Jagiellonian University Medical College, 8 Skawińska Street, 31-066 Krakow, Poland

**Keywords:** amputation, low back pain, exercise, physiotherapy, strengthening, gait

## Abstract

Low back pain is very common condition that often becomes a long-lasting problem in prostheses users after lower limb amputation. The presented study aims to decide the potential benefits of exercise therapy on low back pain among lower limb amputees by using a systematic review. The PICO technique was used to answer the primary issue of this review: Does exercise treatment lessen the prevalence of low back pain in the population of lower limb amputees? Systematic review was conducted in the following databases: Medline-PubMed, EMBASE, Scopus, and Web of Science. Studies up to September 2010 published in English are included. Aim, target population, development and execution strategies, and treatment suggestions were among the data gathered. The primary outcomes of interest were exercise interventions as a therapy for low back pain but only two articles met including criteria. The search was broadened and 21 studies describing biomechanical changes in gait and pelvic-spine posture were analysed. This review indicates that movement therapy is a potential treatment strategy in low back pain among amputees. The major limitation of the study is the very heterogenous group of subjects in terms of amputation level, baseline activity level and comorbidities. We used a procedure that was registered in PROSPERO (CRD42022345556) to perform this systematic review of systematic reviews. There is a necessity of good quality research for concluding a consensus of exercise intervention.

## 1. Introduction

Amputation of the lower limb is a life-changing experience, in which there are alterations in physical and mental well-being [1]. An estimated 1.6 million people were living with the loss of limb in the year 2015; in the United States, 185,000 people undergo an amputation of a lower limb every year. Dysvascular amputations, described as secondary to complications of peripheral arterial disease or diabetes mellitus, are the most common [2]. The leading causes have been reported to vary depending on the region. In many low- and middle-income countries, trauma has been documented as the primary mechanism for limb loss [3]. The multitude of causes and complications of the amputation process make it very difficult to study. Traumatic amputees tend to be younger and better in shape than vascular disease or diabetic amputees. They are also able to make full recoveries from their injury to a point where they can autonomously walk and achieve everyday high-level functioning. Secondary musculoskeletal disability, such as low back pain (LBP), is among the most important sources of additive disability. In the amputee population, between 52–71% of amputees experience LBP [4,5]. The amount is much higher than in the non-amputee population (12–34%) [6]. There have been numerous systematic evaluations of exercises for low back pain but only in the non-amputee population.

Various environmental and individual characteristics have been reported to increase the risk of LBP [7]. Non-specific LBP is defined as pain that is not associated with any identifiable known or particular pathology (e.g., inflammatory, cancerous or infectious process) [8]. Risk factors predisposing to develop LBP include, for example, poor general health, physical, and psychological stress [9]; additionally, low sleep quality leads to the development of musculoskeletal pain [10]. Dailly activates performed after limb loss may differ in biomechanics and can lead to the development of LBP [11]. LBP after amputation may arise due to one or several biomechanical factors simultaneously. These factors can be movement asymmetry, abnormal joint forces, prosthesis type, muscle atrophy [12,13]. Contrasted to other groups of patients such as individuals with hip or knee endoprosthesis or stroke, persons with lower limb amputation (LLA) represent a relatively minor sample with high variability throughout the population. Age, gender, cause of amputation, time after amputation, related comorbidities, and prosthetic care, for example, have all been shown to be varied.

It is well established that using an exercise rehabilitation programme with the non-amputee group of patients with LBP is very beneficial [14,15]. The overall conclusion is that exercises are not helpful or as effective as other therapies for acute low back pain, but they are effective or even more successful than other treatments for chronic low back pain. Exercise can improve extension strength, range of motion (ROM), and functional disability [16]. Several exercises have been recommended to reduce chronic LBP, including lumbar stabilisation training, motor control exercise, core workout, lumbar flexion exercise, and strengthening exercise [17]. Home exercise is a promising way to reduce the absence of availability of special training centres [18]. The rehabilitation approach after amputation presents unique challenges. Clinical advice or guidelines for physicians managing this group of patients with LBP are not yet available. However, there are some articles that present evidence on fundamental mechanisms, prevalence, and management of LBP among people after amputation. The program’s content and individual exercises may be tailored to the level of amputation, residual limb length, and volume of remaining muscle tissue. It is worth noting that most of the research in the literature concerns non-exercise methods for musculoskeletal pain among amputees. 

Evidence-based practise is at the bottom of physiotherapy and is mainly based on systematic literature reviews findings. This review was conducted to find valid information that can help develop targeted interventions and improve rehabilitation programmes among amputees. The main question of this review based on PICO method was: In lower limb amputee population, does exercise therapy reduce prevalence of Low Back Pain? The purpose of this research was to analyse and assess the literature in order to provide evidence statements for the treatment. 

## 2. Materials and Methods

A literature review was completed on the following databases: Medline-PubMed, EMBASE, Scopus, and Web of Science. The description of PICO strategy is presented in Table 1. The primary search terms were ‘lower limb amputation AND low back pain AND exercise’. 

### Search Strategy

((“lower limb amputation” [MeSH Terms] OR “amputation” [All Fields] OR (“above-knee amputation” [All Fields] OR “below-knee amputation” [All Fields] OR “limb loss” [All Fields]) AND (“low back pain” [MeSH Terms] OR (“LBP” [All Fields])) AND (“exercise” [MeSH Terms] OR “exercise” [All Fields] OR (“physical” [All Fields] AND “activity” [All Fields]) OR “physical therapy” OR (“exercise” [All Fields] AND “therapy” [All Fields]) OR “exercise therapy”))) AND ((“2000/01/01” [PDAT]: “2022/12/31” [PDAT] AND English[lang]. The investigation includes English-language papers that were randomised and published between 1 January 2000 to 31 December 2021. The review was performed in agreement with the PRISMA (Preferred Reporting Items for Systematic Reviews and Meta-Analyses) guidelines and PRISMA-P checklist is provided as an additional file. The reviewers were physiotherapist and academic researchers working with amputees’ population daily. 

Inclusion and exclusion criteria were defined for the examination of titles, abstracts, and extensive texts. The review examined works completed and published in English between the years 2000 and 2021. Nonspecific low back pain is defined as persistent low back pain that is not ascribed to an identifiable, recognised specific disease (e.g., infections, cancerous, osteoporosis, ankylosing spondylitis, fracture, inflammatory process, radicular syndrome or cauda equina syndrome) for the purposes of this review [19]. Exercise treatments were described as activities that were planned, systematic, and repeated that result in body movement and energy consumption by engaging skeletal muscles [20]. Post-treatment, short-term (closest to three months), intermediate-term (closest to 6 months), and long-term (closest to 12 months) follow-up.

Exclusion criteria included research published in a language other than English that were unrelated to low back pain in amputation patients. Studies were ruled out if the participants experienced acute or subacute low back pain, or if the circumstance was caused by certain disorders. Bachelor’s, Master’s, and Doctoral theses, as well as Letters to the Editor, Conference reports, and study protocols, were all rejected. Additional exclusion criteria included a lack of properly documented outcomes, availability to full-text papers, and the absence of defined scales for low back pain. The participants in the trials included male and female patients between 18 and 65 years of age who had experienced amputation in the lower extremity. The electronic search was carried out by one reviewer. Two reviewers (AWS and AMB) independently evaluated the titles and abstracts. Two independent reviewers (AWS and AMB) examined these full text papers to determine eligibility. Disagreements were identified and handled between pairs of reviewers and where they required the engagement of a third reviewer. The detailed screening process will be shown in the following Preferred Reporting Items for Systematic Reviews and Meta-Analyses Protocols (PRISMA-P) flow diagram (PROSPERO nr CRD4202234555). In Excel, a data extraction form was specifically created. Information was gathered about general information (e.g., country, healthcare context, publication year, target population and presenting symptoms) (e.g., country, healthcare setting, publication year, target population and presenting symptoms), methods regarding assessed movement activity and implementation (e.g., [strength of] recommendations, any details regarding subgroups of amputees).

Using the Risk-of-Bias 2 tool, which is accessible on the Cochrane platform, the risk-of-bias analysis was carried out independently by two researchers. The analysis looked at the randomization procedure, variations from intended interventions, missing outcome data, measurement of outcome, and choice of the reported result as its five bias domains. Three to seven questions, with possible responses of Yes/Probably Yes/Probably No/No/No information, were included in each domain. The programme evaluated each domain’s and the overall study’s risk of bias based on the responses given.

To evaluate the possibility of bias in nonrandomized studies or therapies, the ROBINS-I analysis was conducted. Confounding participants, interventions, deviations from planned interventions, missing data, measurements, and reported outcomes were the six bias domains that were assessed in the analysis.

## 3. Results

**Figure 1:**
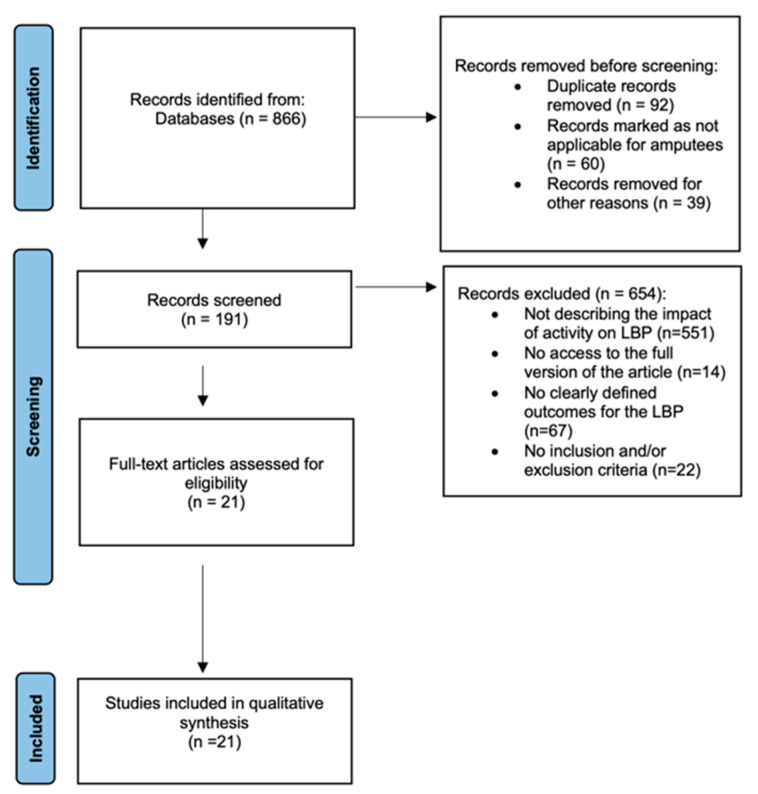
A graph displaying the review (2009 PRISMA-flow diagram stages of the literature).

**Figure 2:**
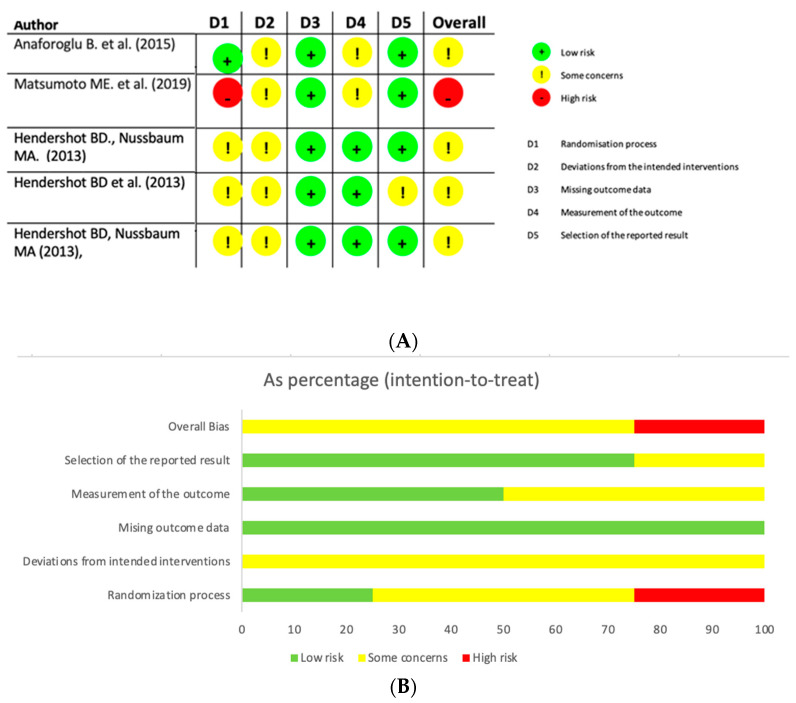
Risk-of-bias analysis (ROB-2) and summary plot of the overall risk of bias. (**A**) RoB-2 analysis [21,22,23,24,25]. (**B**) Summary plot of the overall risk of bias.

Overall, a total of 866 references were found; of those, 675 articles were disregarded due to unrelated title, summaries, and/or language. Finally, 191 were entirely analysed and ultimately only 21 studies incorporated in the review (Figure 1). The ultimate number of publications included in the study was indicated by the risk-of-bias evaluation. The risk-of-bias analysis revealed that the included studies’ quality is either moderate or questionable (Figure 2 and Table 2). 

Critical features of the included and disregarded articles are presented in graphs. 

### 3.1. Exercise-Orientated Rehabilitation Programmes for Low Back Pain in Amputees

Despite many studies available on the low back pain topic and exercise in a non-amputee population, only two studies of this type relevant for amputees were found. The influence of the back school programme in lower limb amputees was examined by Anaforoglu et al. [21]. Twenty men, post-traumatic unilateral transfemoral amputees from the intervention group, performed 10 sessions in two weeks (5 days per week) with back health education and an exercise programme that included practical and theoretical information with individual exercises. Each session lasted about 1 h and was supervised by the physiotherapist. The control group had only a brochure on theoretical info on back health education and exercise pictures. The VAS scale, flexibility and the Oswestry disability index (ODI) were measured before interventions and performed again 1 month and 3 months after treatment. Results show that after 1 month, the pain perception and ODI score decreased significantly in the experimental group (respectively, VAS 34.1 SD 13 in group 1 and 52.8 SD 15.68 in group 2, *p* < 0.001; ODI score 9.55 SD 5.65 in group 1 and 14.85 SD 7.97 in group 2; *p* = 0.03). A similar result was obtained 3 months later (VAS 12.8 SD 8.31; in group 1 and 30.6 SD 10.93 in group 2, *p* < 0.001; ODI score 4.65 SD 3.61 in group 1 and 9.85 SD 5.39 in group 2; *p* = 0.001 [21]. 

Min Kyung Shin et al. [26] examined the influence of lumbar strengthening exercise training in lower limb amputees. A total of 19 subjects unilateral (11 patients) and bilateral (8 patients) posttraumatic amputees were enrolled in the study. There was no control group designed in the study. The exercise programme was conducted twice in a week for 8 weeks, for a sum of 16 sessions, each session was 30 min long and was made up of 14 exercises. Evaluations were performed twice; the first was 1 week before the programme started, while the second was following the 8 week programme. VAS, The Korean version of the Oswestry Disability Index (K-ODI), The Thomas test, and a trunk raising test were used to evaluate changes in participants. Before the project, the mean VAS score was 4.6 SD 2.2, the K-ODI score 12.4 SD 8.2. At the end of the programme, the mean VAS score was 2.6 SD 1.6 and the K-ODI score 11.4 SD 8.2 (*p* < 0.001). Abdominal muscle strength and back extensor strength considerably increased after the programme (Abdominal: 4.4 SD 0.7 before and 4.8 SD 0.6 after, *p* = 0.007; extensor strength before 2.6 SD 0.6 before and 3.5 SD 1.2 after, *p* = 0.007). There was no substantial difference among results in unilateral and bilateral amputees [26]. Study characteristics are showed in Table 3.

### 3.2. Spinopelvic Alignment and Relationship with Low Back Pain among Amputees

Facione et al. [27] examined the spinopelvic alignment in transfemoral amputees using radiologic imaging. 10 male and 2 female patients were classified into two groups: with and without low back pain (LBP). It should be mentioned that 11 subjects used a microprocessor-controlled knee and only one was a mechanical knee. There were 5 subjects in the low back pain group and 7 without it. To analyse postural alignment, biplanar low-dose x-rays of the full spine were made. The researchers found that four subjects with low back pain had an imbalanced sagittal posture (T9 tilt −13^0;^ SD: 4, LBP group and −9^0^ SD:1 non-LBP group; *p* = 0.046). Furthermore, 8 subjects (6 LBP group and 2 non-LBP group) presented an abnormally low value of thoracic kyphosis (TK 26^0^ SD:10, LBP group and 16^0^ SD: 5 non-LBP group; *p*= 0.051). The mean angle TK in the non-LBP group was lower than in the LBP group (*p* = 0.0510). 

A similar study was conducted by Matsumoto et al. [22] in which a relationship only between the lumbar lordosis angle and low back pain was examined using lateral radiological imaging. The authors decided to include 17 transfemoral amputee males, 9 were placed in the LBP group, and 8 in the non-LBP group. Pain levels were characterised using the Chronic Pain Grade questionnaire to rate current pain and intensity, psychological well-being was assessed using the SF-36 Mental Scale and the Roland-Morris Disability Questionnaire was used for 24-item classification of physical disability related to LBP. According to this study, there was no significant difference in the angle of lumbar lordosis (LLA) between two groups with and without LBP (LLA 46.1^0^ SD: 12.4^0^ LBP group and 51.0^0^ SD: 12.6^0^ non-LBP group, *p* = 0.43). Simultaneously there was also no significant difference in sacral inclination angle (SIA) between groups (SIA 38.3^0^ SD:8.7 LBP group and 38.1^0^ SD: 7.5 non-LBP group, *p* = 0.84). Study characteristics presented in Table 4. 

### 3.3. Trunk Kinematics during Standing, Stepping or Sitting Activities

Alterations in trunk-pelvis and lumbar-spine kinematics were measured in three articles by Hendershot et al. [23,24,25]. The first article compares 8 males with unilateral lower leg amputation (transtibial and transfemoral) with 8 male non-amputees as a control group. There was no additional division into the LBP group and the non-LBP group, but those articles highlight changes that might be crucial in the potential development of LBP among amputees. All participants completed the Physical Activity Questionnaire (IPAQ) and their seated balance was assessed using an unstable chair that pivots on a low-friction ball-and-socket joint. Participants performed maximum voluntary contractions (MVC) in the flexion, extension and left/right lateral bending of the trunk. During MVCs, electromyographic (EMG) activities of the bilateral lumbar erector spine, rectus abdominis, and external oblique muscles were recorded [23]. All traditional measures of postural control were significantly higher among participants with lower leg amputation (95% ellipse area, RMS distance, and mean velocity). The RMS distances were higher in the A-P direction for both groups (RMS distance A-P 0.70 cm SD: 0.26 Transtibial; 0.82 cm SD: 0.26 transfemoral and 0.56 cm SD: 0.12 non-amputee group; *p* = 0.015). The mean normalised RMS muscle activity was higher among participants with lower leg amputation in the erector spinae (*p* < 0.0001), rectus abdominis (*p* < 0.0001), and external oblique (*p* = 0.0045). 

In the second study, trunk kinematics and neuromuscular behaviours were compared between people with and without lower leg amputation who performed maximal voluntary standing contractions (MVC) in extension and left/right lateral bending [24]. The same study group that was described before (8 male amputees and 8 male non-amputees) was examined. During MVCs, electromyographic activity was in the same muscle group as before. Furthermore, participants were exposed to horizontal trunk perturbation. Postural displacements were measured with a laser displacement sensor. The main result is that during perturbations, the stiffness of the trunk (TS) and the maximum reflex force (MRF) were significantly lower (TS 13.2 N/mm SD: 2.5 in the non-amputee group; 10.0 N/mm SD: 2.1; *p* = 0.017 and MRF 71.7 N SD:12.3 in the non-amputee group and 55.3 N SD:11.8 in the amputee group; *p* = 0.017). Simultaneously, the effective trunk mass was comparable across groups and perturbation orientations.

The third study had examined flexion-relaxation responses during asymmetric trunk flexion movements [25]. For the third time, the same group took part in the study (8 male amputees and 8 male non-amputees) but this time participants were standing in a fixed structure and movements of the pelvis and lower limbs were further minimised by a fixed pelvic confinement. Twenty-one different movements were performed with the report order of transverse rotation randomised. During each move, subjects flexed forward towards targets until reaching a relaxed, passive hanging position with minimal muscle activity and arms hanging relaxed and then returned to an upright standing position. The main results of these studies showed similar angles of peak lumbar flexion (*p* = 0.26) and peak nEMG values for both flexion (*p* = 0.10) and extension (*p* = 0.33) in sagittal-symmetric movements. In sagittal-asymmetric movements, the maximum lumbar flexion angles decreased significantly (*p* < 0.001) decreased with increasing transverse rotation among participants after lower limb amputation, bilateral similar (all *p* > 0.4). Additionally, during flexion, the peak nEMG values were similar between the groups (*p* = 0.16), transverse rotation angles (*p* = 0.72), and the direction (*p* = 0.24). 

Actis et al. [28] examined subjects with transtibial amputation during the sit-to-stand task. They enrolled 8 participants with lower extremity amputation and 8 without amputation. The dominance of the limb was determined as the leg chosen to kick a ball and everyone completed the Oswestry Low Back Pain Questionnaire. The main task was five sit-to-stand trials with 42 kinematic markers to track feel, shanks, thighs, pelvis, and trunk. Muscle group activation was registered by EMG. The authors developed a musculoskeletal model. Only one individual had LBP that was more severe than minimal, according to the Oswestry questionnaire (30% score). Comparatively to non-amputees, subjects with lower limb amputations showed higher peaks and average L4–L5 compressive loads, peak sit-to-stand motion angles, trunk lateral bending, and axial rotation angular velocities. However, there were no variations in muscle activation that were statistically significant (0.05 < *p* < 0.1). Additionally, it mentions that the amputees generated higher vertical force in the intact limb than the control group due to their greater asymmetry.

Butowicz et al. [29,30] performed two particularly important kinematic investigations. The first analysis looked at how the existence of low back pain influences the joint coordination and balance of the trunk-lower limb during standing. A total of 40 participants were included in this study (23 with LBP and 17 non-LBP amputees). Eight Inertial Measuring Units (sternum, sacrum, bilateral foot, bilateral lower leg, bilateral upper leg) were used to measure the subjects’ standing stillness for 30 s while their eyes were closed and opened. As a result, there was no trunk-hip coordination pattern that indicated the ability to maintain balance while keeping one’s eyes open (Fuzzy Entropy (FE) 0.37 SD:0.08 for the non-LBP group and 0.4 SD:0.10 for the LBP group). With eyes closed, trunk-lower limb joint coordination patterns in the intact limb, such as extension/flexion patterns on the amputated side and flexion/extension patterns on the intact side, predicted FE in the LBP group [29].

The second article by Butowicz et al. [30] was carried out on 32 people with traumatic lower limb amputation and the participants were divided into two groups (LBP-19 and non-LBP-13). The participants were told to maintain their arms crossed and their seat level while sitting in an unsteady chair with their eyes open. Using an 18-camera motion capture system, the three-dimensional trunk-pelvis kinematics were examined with 12 retro-reflective markers. At the same time, EMG data were collected. Centre of Pressure (COP) measures, EMG, and trunk kinematics were compared between groups. As a result we find that there was no main effect of the group on the set of COP-based measures (Wilks’ *Λ* = 0.84, *F*_(10,18)_ = 0.74, *p* = 0.60, *η*^2^ = 0.16) and there was a significant effect on trunk kinematic (Wilks’ *Λ* = 0.46, *F*_(6,19)_ = 3.52, *p* = 0.02, *η*^2^ = 0.54) and muscle activity (Wilks’ *Λ* = 0.69, *F*_(4,15)_ = 3.46, *p* = 0.03, *η*^2^ = 0.31) [30].

The trunk muscle forces and spinal loads during sit-to-stand and stand-to-sit activities were analysed by Shojaei et al. [31]. In the experimental group, there were 10 males with unilateral transfemoral amputation (TFA), and the control group was composed of 10 non-amputees. All participants were military personnel. Participants were asked to perform five consecutive sit-to-stand and reverse movements back to sitting position. There was a force platform under their feet and a 23-camera motion capture system analysing full-body kinematics. The main effects were differences in peak compression (2556 N SD: 731 in TFA; 2208 N SD: 421 in the non-amputee group) and anteroposterior shear forces (373 N SD: 144 in TFA; 221 N SD: 118 in the non-amputee group). The results clearly show that there were larger spinal loads among the amputees during both sit-to-stand and reverse movement. 

Another very important research focused on the kinetic effort of the trunk during ascent and descent of the steps was conducted by Gaffney et al. [32]. In this study, 7 men with unilateral transtibial amputation and 7 men who were able were enrolled. The task for participants was step ascent and descent from a 20 cm platform, while their movement was analysed by 8 near-infrared cameras (63 reflective markers were instrumented to obtain all body kinematics). Peak moments were compared between groups and between limbs during the loading phase. Peak posterior translational trunk moments during vertical thrust of ascent were higher in amputees with severed limbs than in intact limbs in the sagittal plane (*p* = 0.01, g = 1.52 (1.6 2.64)), which can place more strain on the lower back extensor muscles. In the transverse plane, the maximum axial translation moment toward the leading stance foot was higher among amputees when leading with prosthesis or intact limb compared to the healthy group during weight acceptance (*p* < 0.01, g = 2.36 (1.62 5.01) in transtibial amputees; *p* = 0.01, g = 1.47 (1.01 2.94) in the control group). During the descent movement in the sagittal plane, the maximal translational moments of the anterior and posterior trunks were greater among amputees when leading with the intact limb compared to control subjects (*p* < 0.01, g = 1.83 (1.48 3.7) experimental group and *p* = 0.01, g = 1.16 (0.4 3.16) in the control group). In the transverse plane, the peak moment of axial rotation of the trunk toward the leading stance foot was greater among amputees when they step onto the amputated or intact limb compared to the healthy control group (*p* = 0.01, g = 1.13 (0.01 3.78) experimental group *p* = 0.017, g = 1.45 (0.3 4.28)) [32]. 

Murray et al. [33] conducted similar research focussing on biomechanical compensations of the trunk and lower extremities during the step task. The participants in this study were divided into three groups. They included amputees with (n = 10) and without (n = 9) diabetes mellitus as well as an able-bodied control group (n = 11), in contrast to all previous research authors. The steps were 60 cm × 40 cm × 20 cm, put over an adjacent force plate, and the subjects stepped onto them while standing on an associated force plate. Participants in the two-step descent stepped onto the force plate after starting on the step. A total of 63 reflective markers were applied to the participants’ bodies, and the trunk was modelled as a single stiff section. The findings showed that amputees had excessive and asymmetric trunk motion, as well as abnormal joint moments in the lower back and lower limbs when compared to persons with diabetes and healthy people. For all groups, the trunk flexed forward during the entire ascending step in the sagittal plane. Peak trunk flexion among amputees was bilaterally comparable (limb with amputation = 31.8 (7.2); limb with an intact limb = 31.4 (7.5); *p* = 0.8). Amputation patients, but not the diabetic group, showed more trunk flexion than the control group (21.6 SD: 8.60, *p* = 0.0017 for the amputated leg; *p* = 0.02 for the intact limb). In comparison to the diabetes group (*p* = 0.003) and the control group (*p* = 0.001), the transtibial amputee group produced more peak low back extension moments on the severed limb during ascent kinetics. Stepping onto the intact limb during step descent caused the experimental group to experience low back extension moments that were five times higher than those experienced while stepping onto the severed leg. Similar to step ascent, amputees without diabetes displayed a greater hip extension moment when stepping onto an artificial limb (control group *p* = 0.003, 22.6 SD: 5.50 TTA, 16.7 SD: 2.40 DM, 16.2 SD: 4.50). In Table 5, study characteristics are listed.

### 3.4. Trunk Kinematics during Walking Activities

Banks et al. [34] asked a crucially important question. During their study, researchers were analysing if lower back demands can be reduced by improving gait symmetry. The small experimental group consisted only of five amputees and five able-bodied participants. On a treadmill, the subjects were instructed to walk with varying degrees of asymmetry. Using a full-body OpenSim model that was assessed for gait, the L5/S1 vertebral joint forces were estimated for each level of asymmetry. An intriguing finding was that symmetrical gait did not significantly differ in joint forces.

Acasio et al. [35] took into account a larger experimental group (divided into patients experiencing low back pain and those without) and a control all-body group. A total of 35 amputees (19 LBP and 16 non-LBP) and 15 non-amputees walked overground across a 15 m walkway at 1.3 m/s. During the task they were captured with camera motion system. The main effects of the group were observed in the pelvic ROM of the sagittal plane (*p* = 0.018, η^2^ = 0.147), the frontal (*p* < 0.001, η^2^ = 0.327) and the transverse (*p* < 0.001, η^2^ = 0.368) plane, and the thorax ROM (*p* = 0.018, η^2^ = 0.147), frontal (*p* < 0.001, η^2^ = 0.327) and transverse (*p* < 0.001, η^2^ = 0.368) plane (*p* = 0.021, 2 = 0.086) and frontal (*p* = 0.036, 2 = 0.327) plane for lumbar range of motion. However, no pairwise differences were observed between amputees with and without LBP. 

Butowicz et al. [36] researched whether trunk muscle activation patterns were influenced by walking speed. They planned the experiment with eight unilateral amputees and 10 able-bodied control groups. The experimental task was to walk on a 15 m walkway at four different speeds (1, 1.3, 1.6 m/s and self-selected speed). Full-body kinematics were recorded by tracking the location of 51 surface markers, and the erector spine was monitored with electromyographic (EMG). Interesting results showed that there were no differences in the first onset of thoracis erector spinae (TES) during intact stance and any speed between amputees and the control group. However, during an intact stance, amputees activated TES for a higher percentage of the gait cycle and corresponded to increased lateral trunk flexion among amputees. On the contrary to the hypothesis of the researchers, the trunk ROM remained similar at all walking speeds. Fatone et al. [37] presented similar results during the study conducted in 23 amputees divided into two groups with low back pain (n = 12) and without low back pain (n = 11). Participants walked at a self-selected comfortable walking speed along the walkway while videotaping using an 8-camera digital motion analysis system. Again, the pelvic sagittal plane movement patterns were very similar between groups. Only a small increase in anterior pelvic tilt was observed in the LBP group. Based on the sagittal lumbar spine motion, there was no significant difference in the number of patients in the LBP group (W2(1, n = 21) = 0.43; *p* = 0.84; phi = 0.045). A total of 46% of the participants with LBP had the extension pattern, while 54% did not. The research of the kinematics of the lumbar spine during locomotion and low back pain among amputees was expanded by Morgenroth et al. [38] by including a new set of participants. They consisted of 6 healthy, able-bodied control groups and 17 amputees (split into the LBP group of 9 and the non-LBP group of 8). There were three dynamic walking tests run. Once more, there were no appreciable differences between the LBP and non-LBP groups in the sagittal or frontal plane of lumbar spine excursion during locomotion. Only the transverse plane rotation between the LBP and non-LBP groups showed statistical significance.

Golyski and Hendershot [39] questioned earlier studies and devised a plan for observing how the trunk and pelvis move during transient twists in amputees. Twenty spins comprising a 90-degree change in direction to the left and right were executed by eight participants with unilateral lower limb amputations and five able-bodied control groups. A 27-camera motion capture system was used to acquire and analyse full-body kinematic data. The primary findings were that, during spin turns, there were no significant differences in the frequency of any coordination mode during the stance or swing phase (*p* > 0.082). On the other hand, amputees were more likely to exhibit transverse plane pelvis phase coordination during step turns (*p* = 0.036). 

In another study, researchers tried to analyse trunk-pelvic motion during walking with hip strength and knee joint moment among amputees. Butowicz et al. [40] took into account 24 male amputees and 8 able-bodied control groups. No one experienced LBP at the time of the tests. Isometric hip abductor strength was assessed using a hand-held dynamometer as participants walked at a speed of 1.3 metres per second on a 15 m boardwalk. Surprisingly, there was no connection between pelvic or trunk mobility and hip abductor strength. The scientists noticed that amputees had higher trunk lateral flexion and acceleration during stride than the healthy control group. Esposito and Wilken [41] decided to enrol a group of 16 transfemoral amputees and 12 able-bodied control groups and analyse the relationship between pelvic-trunk coordination in LBP. 

Amputees were divided into two groups: 9 with LBP (TFA-LBP) and 7 without LBP (TFA-NP). Participants completed a walking activity on a 20 m walkway and their movement was captured with the motion capture system. The results showed that amputees with and without LBP exhibited coordination of transverse plane movement similar to that of able-bodied (*p* = 0.966). Study characteristics are presented in Table 6. 

## 4. Discussion

The main goal of this review was to analyse differences in physical activity as an approach to treatment of LBP among amputees. The task was impossible to accomplish due to the extremely low number of articles corresponding to this topic. The impact of exercise treatment on LBP in the able-bodied population is widely researched. According to the most recent studies [42,43,44], exercise lowers pain when compared to no therapy, standard care, or a placebo. We can also discover evidence that exercise enhances capacity when compared to therapies such as electrotherapy or education, and it may be more beneficial than hands-on therapist treatment [45]. We also know that the most successful programmes included at least one or two sessions of Pilates or strength exercises each week. Sessions of shorter than 60 min of core-based, strength, or mind–body exercises are also beneficial, as are training programmes of 3 to 9 weeks of Pilates and core-based exercises [16]. When it comes to the amputee population, we can find two research articles that include some types of exercise in the treatment of LBP. Both with satisfactory results for participants who introduced trunk muscle exercise and a back school programme. We decided to include and analyse in this review articles that examine the biomechanics of the trunk, pelvis, and lower extremities that can lead to LBP during various tasks and activities. Although the included studies investigated the origins of LBP in amputees, many of them were of low quality and had significant limitations. The very limited number of trials in physical investigations is a critical limitation, implying that the results may not be typical of the overall population. Furthermore, comparing the amputee population with the able-bodied control group is debatable and makes it difficult to draw convincing conclusions. Another significant restriction is that previous research concentrated on traumatic amputees, although we know that peripheral arterial disease and diabetes are the leading causes of lower extremity amputations [46,47]. Traumatic amputation occurs more frequently in younger, active people who may not yet experience LBP. The authors also paid much less attention to the risk of factors for LBP. Only in four studies was LBP questioned with standardised tools, and in the general population we have a strong correlation between age, gender, abdominal obesity, smoking and the risk of LBP [48]. Furthermore, any current study that takes into account the psychological stress that can lead to LBP among amputees, but we know that limb amputation is an irreversible act that is sudden and emotionally devastating to all patients [49]. This review has identified topics that should be investigated more thoroughly in future studies.

Several trunk and pelvic kinematic patterns occur in amputees that contribute to an additional load on the spine during different activities. Hendershot and Actis [22,23,24,28] examined activities such as sitting and stepping, proving that for people with LLA those tasks are more demanding for muscle and spine joints. Butowicz [30] points out that people with LLA and LBP demonstrated impaired postural control of the trunk. The persistence of LBP among amputees may be the result of neuromuscular adaptation in the proximal structures, but further research is needed to determine the reason behind these increases. Many of the older patients after LLA spend most of their days sitting in a sitting position. Taking into account the results of the presented studies, it seems very reasonable to address stability exercise and strengthening trunk movements as a golden standard of rehabilitation. A similar approach was taken on the able-bodied population resulting in very satisfying outcomes [50,51]. To maintain mobility after LLA, patients must adopt movement compensations to account for loss of knee or ankle function in the amputated limb. Reduced trunk muscle activation and increased intramuscular fat may be potential intervention targets after LLA [52]. Additionally, the intermuscular fat content of residual limb endings rises over time [53]. Murray [33] was the only author to take into account amputees with diabetes and performed stepping tasks among the experimental and control groups. Half of people with diabetes have nerve damage that influences their control of the neuromotor muscles. Murray’s study shows that people with LLA and diabetes exhibited excessive and asymmetric trunk motion, which was assisted by an asymmetric load on the low back. There are many good quality articles that show a significant improvement in pain and quality of life after exercise therapy among patients with diabetes [54,55]. 

Walking is a highly repetitive task that exposes people with LLA to large alternations of spinal loads. To reduce the stress on the weaker hip abductor muscles on the side of the residual limb, people with LLA prefer to engage in considerably increased lateral bending towards the prosthetic limb during single-limb stance and double-limb support. Greater value in the intact limb imply that it is important in maintaining stability and optimising body progression throughout various tasks [56]. There is moderate to strong evidence that chronic LBP patients have different walking gaits than healthy controls in the able-bodied population [57]. Fatone and Morgenroth [37,38] tried to assess lumbar and pelvic kinematics during gait in transfemoral amputees. Both studies concluded that differences in lumbar and thoracic motion do not appear to be independently related to LBP. Furthermore, some studies that investigated the mechanics of altered amputees did not report the prevalence of LBP, so it is challenging to draw mechanical conclusions that relate altered gait to LBP. Esposito [41] concluded that individuals with LLA with and without LBP had similar transverse plane movement coordination to able-bodied people and that only amputees could have found the increases in speed problematic enough to achieve a change in transverse plane coordination. Naturally, it could lead to the statement that we should exercise gait patterns and improve gait symmetry in the LLA population, but Banks [34] tried to answer this theory in his research. The study revealed that training amputees to walk more symmetrically may not decrease low back demands since they already locomote at a desired degree of asymmetry, which clearly reduces L5/S1 joint loads. Exercise therapy using, for example, the Pilates method can improve weight discharge in gait and reduce LBP [58]. 

Gait and posture bring us to the use of a prosthetic leg during walking and other activities of daily living. The purpose of prosthetic design is to replicate the anatomy and function of the missing limb. [59]. The studies presented in this review did not take this into account because the designed groups were mainly composed of high-functioning people with LLA who often used electronic knee prostheses. Many prosthetic parameters, including limb weight, number of artificial joints, prosthetic length, and prosthetic attachment, may contribute to the beginning of LBP in the general population of patients with LLA [60]. Additionally, the weight of the prosthesis device itself demands more energy of the low back, hip muscles, and core musculature. This brings us to the conclusion that core muscle strength and back muscle endurance are needed to walk with a prosthesis efficiently. The benefit would be to focus on stabilisation exercise therapy. Resistance training (especially in the core and lumbar extensor muscles) may enhance gait metrics, according to emerging research [61,62]. 

This systematic review’s limitations may have introduced some possible biases. The low confidence of evidence is a result of some of the included reviews’ poor methodological quality and the RCTs that underlie them. The overall number of participants was low for the majority of outcomes and time periods. High levels of overlap were produced by the inclusion of the same RCTs in several evaluations, and the inconsistent findings of the review authors’ methodological quality assessments of some RCTs are further a cause for concern. 

LBP is one of the most frequent conditions for which people seek out primary care services from a physiotherapist or general practitioner. For this condition, it’s crucial to offer precise, efficient management. To support the professions of physical medicine and rehabilitation, physical therapy, and prosthetics in achieving the objective of standardisation in the care of patients with LLA, evidence-based practise must go beyond just using outcome measures as evaluation tools. Understanding the patient’s response to therapy (improvement, no change, decrease) enables doctors to provide care more quickly, lowering overall rehabilitation time and increasing patient outcomes [63]. 

### Potential Benefits of Exercise-Based Rehabilitation in People with LLA

Current research in various therapeutic groups supports the advantages of strengthening, balance, and stability training on back pain. The goal of the exercise programme should be to improve muscle tissue quality, strength, endurance, and balance, as well as to reduce movement asymmetries during locomotion and weight-bearing duties. Amputees suffering from muscular atrophy and fibre type shifting may benefit directly from improvements in muscle growth, strength, and endurance. Resistance exercise has been shown to dramatically decrease discomfort [64]. Resistance training causes morphological modifications in skeletal muscle such as muscle fibre hypertrophy and cross-sectional area, as well as an increase in the amount of connective tissue surrounding muscle fibres [65]. 

Improved strength, endurance, and stability of knee extensors and flexors in transtibial LLA may allow for improved stability of the intact limb during stance and propulsive forces provided by the limb during walking. Other advantages include better walking endurance, confidence in gait speed, and greater independence in everyday tasks [66]. Lunges, plank variants, and multidirectional reaching increase control and strength of the core, hip flexors, and adductors, which help to stabilise the spine. Core and balance training is critical for improving distal mobility and the generation of strong limb motions [67]. Following long-term general prescription recommendations, regular resistance exercise participation is suggested to address physiological and mechanical difficulties that support low back discomfort.

Exercise therapy may or may not be the best solution depending on a number of factors, including the patient’s preferences and values, the physiotherapist’s clinical skills, and the results of the study. This is in accordance with the principles of evidence-based treatments. 

## 5. Conclusions

Our findings show that intervention groups employing an exercise intervention had considerably lower chronic low back pain than other therapies, but there are not enough trials to produce evidence-based practice. Exercise therapies, such as core strengthening and stability exercises, have the potential to significantly reduce low back discomfort in amputees. For future studies, better methodological research that compares the different amputee groups (TFA/TTA/able body/LBP/non-LBP/unilateral/bilateral) systemically in terms of personal factors influencing LBP, their anatomical structures, and their biomechanical movements under various conditions is needed for a better understanding of the mechanisms of LBP among people with LLA.

Limitations:

- The results of this systematic review may have been biased because the only study language used in the included RCTs was English and the study sample sizes were small.
- The exercise therapy was inconsistent throughout the included RCTs, and the controls’ treatments varied as well.
- High levels of heterogeneity within relatively small segments of the literature may have had an impact on the validity of the review.
- The risk of bias of presented studies was moderate or debatable.

## Figures and Tables

**Table 1:** Description of PICO strategy.

PICO Elements	Keywords	Search Terms	Search Strategies
P (Population)	Lower limb amputees	Lower limb amputation	Above-knee amputation OR below-knee amputation OR limb loss
I (Intervention)	Exercise therapy	Exercise therapy	Stability exercises OR strengthening exercises OR core stability exercise OR stretching OR Pilates
C (Comparison)	Manual Therapy or no therapy or electrotherapy		
O (Outcome)	Reduce prevalence of low back pain	Low back pain	Low back pain

**Table 2:** ROBINS analysis.

Author	Bias Due to Confounding	Bias in Selection of Participants into the Study	Bias Due to Missing Data	Bias in Measurement of Outcomes	Bias in Selection of the Reported Result	Overall Bias
Shin, 2018 [26]	Serious	Low	Low	Moderate	Low	Moderate
Facione, 2019 [27]	Serious	Moderate	Moderate	Moderate	Serious	Serious
Actis, 2017 [28]	Serious	Moderate	Moderate	Moderate	Serious	Serious
Butowicz, 2019 [29]	Moderate	Moderate	Low	Low	Low	Moderate
Butowicz, 2019 [30]	Moderate	Moderate	Low	Moderate	Low	Moderate
Shojaei, 2019 [31]	Serious	Moderate	Low	Moderate	Serious	Serious
Gaffney, 2017 [32]	Serious	Moderate	Moderate	Low	Serious	Serious
Murray, 2017 [33]	Serious	Moderate	Moderate	Serious	Serious	Serious
Banks, 2022 [34]	Serious	Serious	Low	Moderate	Serious	Serious
Acasio, 2022 [35]	Serious	Low	Low	Moderate	Moderate	Moderate
Butowicz, 2018 [36]	Modarate	Low	Modarate	Modarate	Low	Modarate
Fatone, 2015 [37]	Serious	Moderate	Moderate	Serious	Moderate	Serious
Morgenroth, 2010 [38]	Serious	Moderate	Moderate	Moderate	No info	Serious
Golyski, 2018 [39]	Serious	Low	Low	Moderate	Serious	Moderate
Butowicz, 2020 [40]	Moderate	Low	Moderate	Serious	Low	Moderate
Esposito, 2014 [41]	Moderate	Serious	Low	Moderate	Serious	Serious

**Table 3:** Exercise-orientated study characteristics.

First Author (Year), Country	Title of Article	Main Objective	Sample Demographics	Research Tool	Main Results
Anaforoglu B. et al. (2015), Turkey [21]	The effectiveness of a back school program in lower limb amputees: a randomized controlled study	Assessment of a back school program in lower limb amputees	Con. gr. = 20 Exp. gr = 20	ODI VAS Spinal flexibility measurements	There was a significant reduction of VAS and ODI scores after 3 months. Back health education has positive short-term effects.
Shin MK. et al. (2018), Korea [26].	Effects of Lumbar Strengthening Exercise in Lower-Limb Amputees with Chronic Low Back Pain	Analyse the effect of lumbar strengthening exercise in lower-limb amputees with chronic low back pain after an 8 week training program.	Exp. gr. = 19 Con. gr = non	ODI VAS Iliopsoas length Abdominal and back extensor muscle strength, back extensor endurance (isokinetic dynamometer)	Abdominal muscle and back extensor strength improved significantly after 8 weeks. The VAS decreased significantly after treatment. The peak torque and total work of trunk flexors and extensors increased significantly.

ODI—Oswestry Disability Index, VAS—visual analogue scale

**Table 4:** Spinopelvic alignment study characteristics.

First Author (Year), Country	Title of Article	Main Objective	Sample Demographics	Research Tool	Main Results
Facione J. et al. (2019), France [27]	Spinopelvic sagittal alignment of patients with transfemoral amputation	Describe the spinopelvic sagittal alignment in transfemoral amputees from radiologic study of the spine.	LBP gr = 5 Non-LBP gr. = 7	X-rays with 3-D	Altered sagittal balance parameters were found in some subjects, maybe more frequently in LBP group. A low TK angle seems to be associated with the absence of LBP.
Matsumoto ME. et al. (2019), USA [22]	The relationship between lumbar lordosis angle and low back pain in individuals with transfemoral amputation.	Determine whether the extent of lumbar lordosis is associated with LBP in transfemoral amputees	LBP gr. = 9 Non-LBP gr. = 8	x-ray CPG MHS SF-36 24-item R-MDQ	No difference in LLA or SIA between those with and without LBP. Increased LLA is not associated with LBO in this population.

CPG—Chronic Pain Grade, MHS SF-36—Mental Health Scale of the SF-36, 24-item R-MDQ—24-item Roland-Morris Disability Questionnaire, LLA—lumbar lordosis angle, SIA—sacral inclination angle.

**Table 5:** Trunk kinematics during different activities study characteristics.

First Author (Year), Country	Title of Article	Main Objective	Sample Demographics	Research Tool	Main Results
Hendershot BD., Nussbaum MA. (2013), USA [23]	Persons with lower-limb amputation have impaired trunk postural control while maintaining seated balance.	Investigate trunk postural control among persons after limb amputation during seated stability task	Exp. gr. (amputees) = 8 Ctr. gr. = 8	IPAQ, Unstable chair, MVC in trunk flexion, extension, left/right bending, EMG bilateral lumbar, erector spinae, rectus abdominis, external oblique muscles.	All measures of postural control were significantly larger among LLA. RMS distances were larger in the A-p direction for both groups. Mean normalized RMS muscle activity were larger among LLA.
Hendershot BD et al. (2013), USA [24]	Persons with unilateral lower-limb amputation have altered and asymmetric trunk mechanical and neuromuscular behaviours estimated using multidirectional trunk perturbations.	Use of multidirectional trunk perturbations to investigate the effects of LLA on several aspects of trunk mechanical and neuromuscular behaviours.	Exp. gr. (amputees) = 8 Ctr. gr. = 8	MVC in trunk extension and left/right lateral bending with pelvis restrained, EMG bilateral lumbar, erector spinae, external oblique muscles.	During A-P perturbations, trunk stiffness and MRF were significantly lower among participants with LLA compared to non-amputees.
Hendershot BD, Nussbaum MA (2013), USA [25]	Altered flexion-relaxation responses exist during asymmetric trunk flexion movements among persons with unilateral lower-limb amputation	Controlled trunk flexion-extension movements to investigate the effects of LLA on active-passive load sharing mechanism in the low back.	Exp. gr. (amputees) = 8 Ctr. gr. = 8	EMG bilateral lumbar, erector spinae, rectus abdominis, external oblique muscles, MVC in trunk extension and left/right lateral bending.	Decreased and asymmetric passive contributions to trunk movements were compensated with increases in the magnitude and duration of active trunk muscle response
Actis JA. et al. (2017), USA [28]	Lumbar loads and trunk kinematics in people with a transtibial amputation during sit-to-stand.	Characterize the low back biomechanics of people with and without unilateral TTA during sit-to-stand using muscoskeletal modelling.	Exp. gr. (amputees) = 8 Ctr. gr. = 8	MOLBPQ, motion capture system, EMG, GRF	Amputees had greater peak and average L4–L5 loading in compression compared to control group, with peak loading appearing after lift-off from chair. Muscle forces were not significantly different between groups.
Butowicz CM. et al. (2019), USA [29]	Low back pain influences trunk-lower limb joint coordination and balance control during standing in persons with lower limb loss	Determine the influence of coordination between the trunk and lower limbs joints on balance control during standing among persons with LLA with and without LBP	Exp. gr. (LLA with LBP) = 23 Ctr. gr. (LLA, non-LBP) = 17	VAS, standing with eyes open and closed while wearing IMUs, FE	No combination of trunk-lower limb joint coordination predicted FE in eyes open condition in either group. Positive trunk-hip coordination on the intact limb and negative trunk-hip on the prosthetic side limb predicted FE only in LBP group.
Butowicz CM. et al. (2019), USA [30]	Chronic low back pain influences trunk neuromuscular control during unstable sitting among persons with lower-limb loss	Investigate the potential role of impaired trunk postural control among persons with LLA and LBP using an unstable sitting paradigm.	Exp. gr. (LLA with LBP) = 18 Ctr. gr. (LLA, non-LBP) = 13	ODI, VAS, motion capture system, EMG bilaterally from TES and LES.	Persons with LLA and LBP shows impaired trunk postural control compared to those without LBP, as evidenced by reduced local dynamic trunk stability and greater trunk motion during unstable sitting.
Shojaei I. et al. (2019), USA [31]	Trunk muscle forces and spinal loads in persons with unilateral transfemoral amputation during sit-to-stand and stand-to-sit activities.	Comparison of trunk muscle forces and spinal loads between with LLA and without during sit-to-stand and stand-to-sit activities.	Exp. gr. (amputees) = 10 Ctr. gr. = 10	Force platform, motion capture system	The peak compression force and antero-posterior shear forces were, respectively, larger in persons with LBP vs. non-LBP
Gaffney BMM. Et al. (2017), UDA [32]	Trunk kinetic effort during step ascent and descent in patients with transtibial amputation using angular momentum separation	Evaluate trunk compensations and the associated kinetic effort needed for patients with unilateral TTA to perform step ascent and descent tasks.	Exp. gr. (amputees) = 7 Ctr. gr. = 7	Motion capture system	It remains unclear what level of trunk movement compensation can be used to compensate for the loss of active plantarflexion without having potential adverse effects through increased low back demand.
Murray AM. Et al. (2017), USA [33]	Biomechanical compensations of the trunk and lower extremities during stepping tasks after unilateral transtibial amputation	Identify biomechanical compensations of the trunk, hip and knee during step ascent and step descent tasks in individuals TTA with DM, without DM and healthy control group.	Exp. gr. (amputees no DM) = 9 Exp. gr. (amputees with DM) = 10 Ctr. gr. = 11	Force plates, motion capture system,	During step up and down, persons with DM and TTA exhibited asymmetrical and excessive trunk motion, which was accompanied by asymmetrical loading of low back.

IPAQ—Physical Activity Questionnaire, MVC—Maximal Voluntary Contraction, EMG—electromyographic, LLA—lower limb amputees, RMS—Root mean Square, MRF—maximal reflex force, TTA—transtibial amputation, MOLBPQ—Modified Oswestry Low Back Pain Questionnaire, GRF—Ground Reaction Force, LBP—Low Back Pain, VAS—Visual Analog Scale, IMU—Inertial Sensor Module, FE—Fuzzy Entropy, ODI—Oswestry Disability Index, TES—Thoracic Erector Spinae, LES—Lumbar Erector Spinae, COP—Centre of Pressure, TTA—transtibial amputation, DM—Diabetes Mellitus.

**Table 6:** Trunk kinematics during walking activities study characteristics.

First Author (Year), Country	Title of Article	Main Objective	Sample Demographics	Research Tool	Main Results
Banks JJ. et al. (2022), USA [34]	Are lower back demands reduced by improving gait symmetry in unilateral transtibial amputees?	Examination of pre-existing dataset to explore whether L5/S1 vertebral forces in people with LLA can be improved with better symmetry.	Exp. gr. (amputees) = 8 Ctr. gr. = 8	Motion Capture System	Results challenge the premise that restoring symmetric gait in people with LLA will reduce risk of lower back pain.
Acasio JC. et al. (2022), USA [35]	Trunk muscle forces and spinal loads while walking in persons with lower limb amputation: Influences of chronic low back pain.	Evaluation of trunk-pelvic motion, corresponding trunk muscle forces, and spinal loads among persons with LLA and comparison between those with LBP versus those without LBP.	Exp. gr. (amputees LBP) = 19 Exp. gr. (amputees non-LBP) = 16 Ctr. gr. = 15	Motion Capture System, Visual 3D	Despite differences in trunk and pelvis kinematics between LLA with LBP and without LBP, trunk muscle forces and spinal loads were similar between groups.
Butowicz CM. et al. (2018), USA [36]	Trunk muscle activation patterns during walking among persons with lower limb loss: Influences of walking speed	Determine trunk muscle activation patterns and corresponding trunk-pelvic segmental coordination in persons with LLA.	Exp. gr. (amputees) = 8 Ctr. gr. = 10	ODI, Motion Capture System, EMG activities of the erector spinae,	People with LLA demonstrate altered activation of posterior trunk muscle compared to able-bodied control group.
Fatone S. et al. (2015), USA, Australia [37]	Pelvic and Spinal Motion During Walking in Persons With Transfemoral Amputation With and Without Low Back Pain	Investigate differences in spinal kinematics during walking in persons with TFA with LBP and without LBP.	Exp. gr. (TFA with LBP) = 12 Ctr. gr. (TFA non-LBP) = 11	SCS, Motion Capture System, Visual 3D	Using a regional spine model some differences in sagittal and transverse lumbar spine kinematic were observed between groups but not possible to confirm these finding was related to the presence of LBP.
Morgenroth DC. et al. (2010), USA [38]	The relationship between lumbar spine kinematics during gait and low-back pain in transfemoral amputees.	Investigate the differences in lumbar spine kinematics between TFA with and without LBP	Exp. gr. (amputees LBP) = 9 Exp. gr. (amputees non-LBP) = 8 Ctr. gr. = 6	CPGQ, MHS of SF-36, DAAS, 24-item RMBPQS, CCMS, Motion Capture System	There were no significant differences in sagittal or frontal plane lumbar spine between groups. The association between increased transverse plane motion in the lumbar spine during gait and LBP was found.
Golyski PR. And Hendershot BD. (2018), USA [39]	Trunk and pelvic dynamics during transient turns among individuals with unilateral traumatic lower limb amputation.	Characterize proximal compensations using inter-segmental momenta and coordination during transient turns among subjects with LLA	Exp. gr. (amputees) = 8 Ctr. gr. = 5	VAS, Motion Capture System	Differences in the frequencies of inter-segmental coordination, trunk-pelvis ROM and segmental momenta across levels of amputation, depending on the plane and method of turn applied were found.
Butowicz CM. et al. (2020), USA [40]	Relationships between mediolateral trunk-pelvic motion, hip strength, and knee joint moments during gait among persons with lower limb amputation	Determine the relationship between knee joint loading, mediolateral trunk and pelvic motion and bilateral hip abductors strength during gait among people with LLA	Exp. gr. (amputees) = 24 Ctr. gr. = 8	Dynamometr, Motion Capture System	There were no group differences in hip strengths, peak knee adduction moment or pelvis acceleration between groups. There was increase in trunk lateral flexion and acceleration during gait.
Russell Esposito E. and Wilken JM. (2014), USA [41]	The relationship between pelvis-trunk coordination and low back pain in individuals with transfemoral amputations.	Analyse how pelvis-trunk kinematics, coordination and coordination variability differs among persons with TFA with and without LBP	Exp. gr. TFA LBP) = 9 Exp. gr. (TFA non-LBP) = 7 Ctr. gr. = 12	PEQ, Motion Capture System	Persons with TFA with and without LBP exhibited similar transverse plane movement coordination to able bodied.

LLA—lower limp amputation, ODI—Oswestry Disability Index, TFA—transfemoral amputation, SCS—Socket Comfort Score, CPGQ—Chronic Pain Grade Questionnaire, MHS—Mental Health Score, DAAS—Day Amputee Activity Score, RMBPQS—Roland Morris Back Pain Questionnaire Scale, CCMS—Charlson Co-Morbidity Score, VAS—Visual Analog Scale, PEQ—Prosthetic Evaluation Questionnaire.

## Data Availability

The data presented in this study are available on request from the corresponding author.
